# Response to: Female Genital Mutilation and Obstetric Outcomes: Flawed Systematic Review and Meta-Analysis Does Not Accurately Reflect the Available Evidence

**DOI:** 10.1155/2016/8376260

**Published:** 2016-08-15

**Authors:** Rigmor C. Berg, Jan Odgaard-Jensen, Atle Fretheim, Vigdis Underland, Gunn Vist

**Affiliations:** Norwegian Knowledge Centre for the Health Services, P.O. Box 7004, St. Olavs Plass, 0130 Oslo, Norway

This is our response to Meirik, Banks, Farley, Akande, Bathija, and Ali's letter to the editor from March 2015 [1], where they comment on our paper “The Obstetric Consequences of Female Genital Mutilation/Cutting: A Systematic Review and Meta-Analysis” [2] and a linked technical report. Unfortunately, we were not informed about their letter; hence our response was late.

We thank Meirik et al. for their careful reading of our paper and technical report. They raise two concerns: (1) our inclusion of results from different research designs (mixing prospective and retrospectively collected data) and (2) our use of unadjusted effect estimates.

In fact, we discussed these concerns directly with Meirik et al. after we had published our systematic review. We acknowledged their concerns and subsequently carried out an update of our analyses limited to prospective studies and adjusted estimates. In the reanalysis, published in this journal last year, we found that “meta-analyses based on adjusted estimates, with or without data from retrospective studies, consistently pointed in the same direction as our earlier findings. There were only small differences in the sizes or the level of statistical significance.” That is, these updated results demonstrate, contrary to Meirik and associates' assumptions, that adjustment did not change the conclusions.

Thus, we maintain that there is convincing evidence that FGM/C is associated with an increased risk of obstetric complications, but that the available evidence does not allow for firm conclusions about how strong this relationship is.
